# 

**Published:** 1908-05-23

**Authors:** 


					J^ay 23, 1908. THE HOSPITAL.
CONTENTS.
SauHSJi^y's Children
189
,y s CnHdren ...
AnnotaM?iiea(1 p?isonin&
190
Annotations- *?lsonln&
^kpTiw?,0^ ?a?> Quackery?The Royal Society and Cancer Re-
IWediooi ? > Remarkable Career-The Infants' Binder
191
IVXedinai ^ .JlLmarKa&Ie Career?The Infants Binder
Movement
192
Clinics?
ana xuovement
A Ara??^Car,liac Arrhythmia.
By Leonard Williams, M.D.,
]yj q -cxixu^tiimia. uy i^eonara >v imams, m.u.
195
Special Article-
Xttedi?e' s Pancreatic Reaction in the Urine
196
Medicine 1
b ancreatic Reaction in the Urine
Urotror/ Co?P^?s.sed Treatment of Diabetic Gangrene ..
199
Boint:
ijrrrf ~ . : i''V bea Alr treatment ot Uiabetic Gangrene
lntn <E.n? 111 Biliar-V and Pancreatic Inflammations ...
200
pot in umary i
so,ts,^..s^rFery-
and Pancreatic Inflammations
!?'"e Special Aneurysn
201
Tho c ^ r ?-"eurysiiis
urfflcal Significance of Vomiting
202
Diseases of Children?
Sudden Death in Infectious Diseases
203
Obstetrics?
The Examination of Patients during Pregnancy
204
Tropical Diseases?
Filariasis?II.
205
Therapeutics -
Prescriptions and Dispensing ...
2C6
"The Hospital" medical Book Supplement.?
No. VII
207
Hospital Administration?
The Evelina Hospital for Sick Children
211
Uurslng- Administration?
The Bedding and Linen department?IV.
215
Editor's I>etter-Box
216
The Graduates' IVXedieal Schools  216

				

